# Expanding Human Breg for Cellular Therapy in Transplantation: Time for Translation

**DOI:** 10.1097/TP.0000000000005243

**Published:** 2024-10-23

**Authors:** Adam McNee, Ananya Kannan, Patrick Jull, Sushma Shankar

**Affiliations:** 1 Nuffield Department of Surgical Sciences, University of Oxford, John Radcliffe Hospital, Headington, Oxford, United Kingdom.; 2 Oxford University Medical School, John Radcliffe Hospital, Headington, Oxford, United Kingdom.

## Abstract

Regulatory B cells (Breg) are instrumental in protecting allografts in transplantation. Breg signatures are identified in operationally tolerant human kidney transplant recipients and can predict organ survival and acute rejection. Animal models of transplantation and autoimmunity support the use of Breg as an adoptive cellular therapy. Detailed mechanistic studies have identified multiple signaling pathways utilized by Breg in their induction, expansion, and downstream function. These preclinical studies provide the guiding principles, which will inform protocols by which to expand this crucial immunoregulatory population before clinical use. There is an urgent need for novel therapies to improve long-term transplant outcomes and to minimize immunosuppression-related morbidity including life-threatening infection and cancer. Systematic evaluation of the signals, which drive Breg expansion, will be key to transforming the as of yet unharnessed potential of this potent immunoregulatory cell. In this review, we explore the potential avenues of translating Breg subsets from cell culture at the laboratory bench to cell therapy at the patient’s bedside. We will discuss the standardization of Breg phenotypes to aid in precursor population selection and quality control of a Breg-cell therapy product. We will evaluate avenues by which to optimize protocols to drive human Breg expansion to levels sufficient for cellular therapy. Finally, we will examine the steps required in process development including scalable culture systems and quality control measures to deliver a viable Breg-cell therapy product for administration to a transplant recipient.

## Graphical abstract

**Figure:**
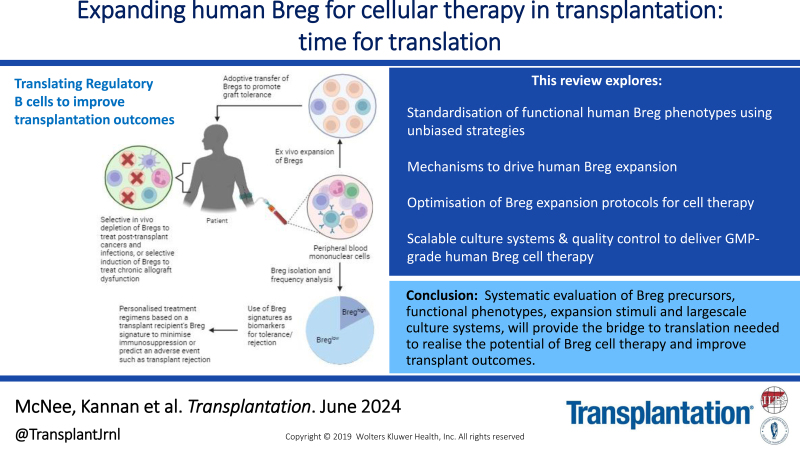

## INTRODUCTION

The importance of regulatory B cells (Breg) in transplantation has become evident over the last decade. Breg control immune responses in animal models of transplantation,^1,2^ Breg signatures are identified in operationally tolerant human kidney transplant recipients^3,4^ and Breg frequency can predict acute organ rejection and allograft function.^5^ Current post-transplant immunosuppressive regimens result in side effects including life-threatening infection (31% of deaths after 1 y) and cancer (7% of deaths), with little improvement in long-term graft attrition compared with previous eras: 25% of all transplants are lost at 5 y.^6^ Novel therapeutic strategies are urgently required, and the application of Breg cellular therapy to promote operational tolerance is an exciting prospect. However, controversy remains regarding whether Breg develop from specific pro-Breg lineages or whether broader populations of B cells can adopt regulatory function after receiving appropriate signals, often to express the immunosuppressive cytokine, Interleukin-10 (IL-10).^7,8^ No unique phenotypic markers have been identified to define a human Breg, which exist across B-cell subsets of increasing maturity.^8-10^ Moreover, the rarity of human Breg and phenotypic and functional disparities between human and mouse Breg are limiting clinical translation.^7,8^

In this review, we explore the potential routes of translating Breg subsets from cell culture to cell therapy. We will discuss potential standardization of Breg phenotypes to aid in precursor population selection and quality control of Breg-cell therapy products. We will evaluate avenues by which to optimize protocols to drive human Breg expansion to levels sufficient for cellular therapy. Finally, we will examine the steps required in process development to deliver a Breg-cell therapy product for administration to a patient.

## IDENTIFYING HUMAN BREG SIGNATURES

Although spontaneous IL-10 secretion by “natural Breg” has been reported,^11,12^ the majority of Breg identified in mice and humans require stimulation to exhibit immunosuppressive function. Multiple stimulation and culture conditions have generated a spectrum of Breg phenotypes. These phenotypes have been extensively described elsewhere by ourselves and others^7,8^ and are not the focus of this review. IL-10^+^ Breg are generally described as being “enriched,” rather than being uniquely expressed within phenotypically distinct subsets. However, proportions of both IL-10^-^ and IL-10^+^ B cells within these subsets are thought to be proinflammatory.^9^ Moreover, the assessment of Breg phenotype based on cytokine expression after a period of ex vivo stimulation can present major limitations. Cell phenotype may alter depending on factors including the duration and nature of stimulation, as well as potential confounding survival pressures within in vitro cocultures, which may reconfigure the distribution of B-cell subsets. Consequently, Breg are more reliably defined by their suppressive ability rather than by phenotype. The identification of accurate human Breg signatures will inform multiple aspects of clinical application: elucidation of optimal precursor populations from which to expand Breg-cellular therapy; rapid and accurate assessment of a Breg-cell product following Good Manufacturing Practice (GMP)–based expansion; in vivo assessment of Breg-cell therapy once administered to the patient; accurate biomarker discovery within endogenous Breg to predict allograft function and to direct immunosuppression minimization or escalation to improve long-term transplant outcomes.

### Previous Strategies to Identify a Breg-specific Marker

**FIGURE 1.**
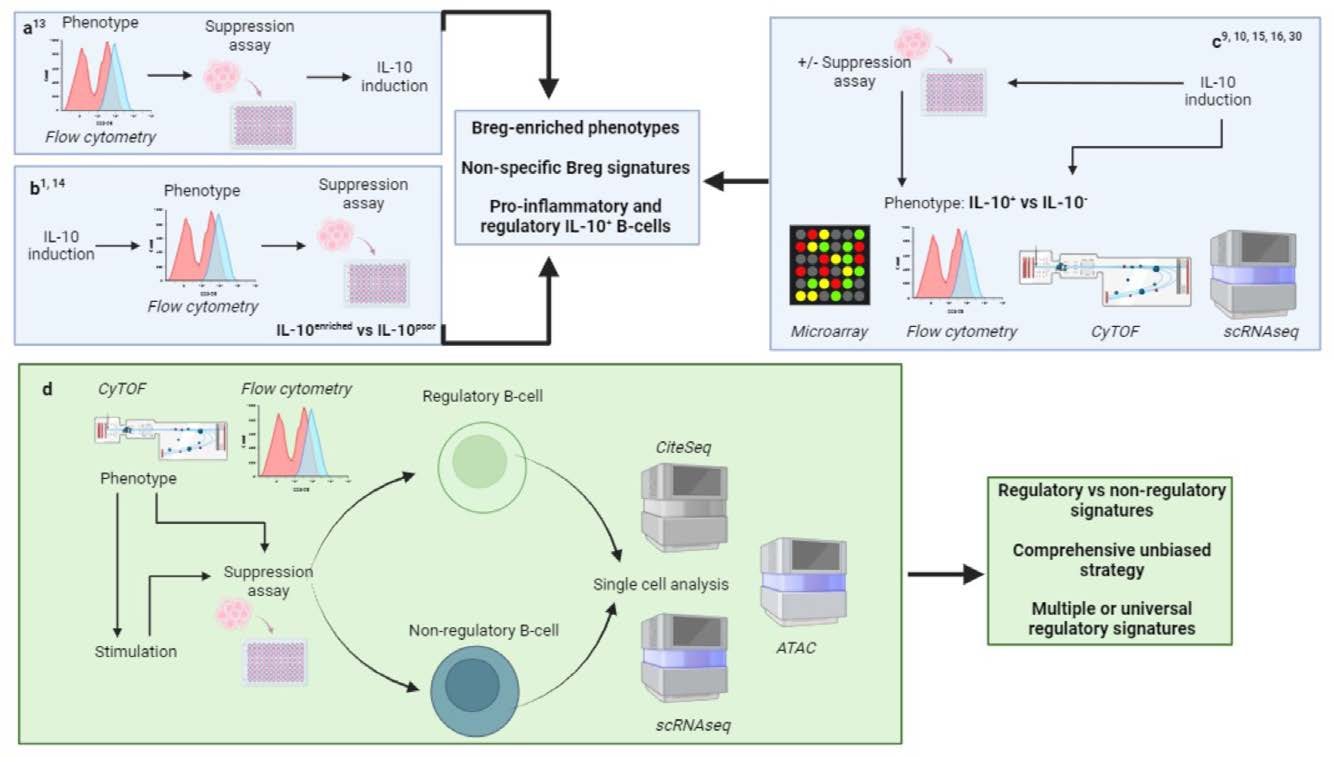
Strategies to identify a Breg-specific marker. Earlier approaches have identified Breg-enriched B-cell subsets by using predefined phenotypic markers and assessing immunoregulatory function and/or IL-10 expression of these subpopulations (A), inducing IL-10 expression by B cells, phenotyping B-cell populations using predefined markers into IL-10^enriched^ vs IL-10^poor^ B-cell subsets using flow cytometry and then assessing immunoregulatory function (B), and inducing IL-10 expression by B cells and assessing proteomic and transcriptional profiles of IL-10^+^ vs IL-10^−^ B cells (C). D, Alternative approaches to identify specific Breg signatures may involve defining human Breg principally by their immunoregulatory function and then evaluating transcriptional and proteomic profiles using non-directed methods. Key comparisons will include nonregulatory B-cell populations. If Breg require stimulation before exerting suppressive function, comparative nonregulatory B-cell populations will require the same/similar modes of stimulation as Breg before analysis. This alternative strategy maybe more able to reveal unique immunoregulatory signatures within human B cells. Figure generated in Biorender.

Historically, attempts to characterize human Breg have predominantly taken a directed approach. Strategies are based on stratifying B cells using predefined phenotypic markers usually including immunosuppressive cytokine IL-10, by methods such as flow cytometry, microarray, CyTOF or cell sorting, and immunoregulatory function then assessed (Figure 1A–C).^13,14^ The majority of human Breg appear in part to depend on IL-10 to exert immunosuppressive function. To detect IL-10 expression or production, B cells require stimulation. It is commonly assumed that IL-10^+^ human B cells within predefined B-cell subsets with immunoregulatory function represent a discrete Breg population. The technologies used to stratify B-cell subsets usually target a small range of predefined phenotypic markers to identify B-cell phenotypes.

There are several limitations to this approach. A wide range of stimulatory conditions may have contributed to the huge diversity of human Breg phenotypes, magnitude of IL-10 expression, and mechanisms of action.^7^ It is unclear whether the same underlying molecular mechanisms drive immunoregulation mediated by these apparently diverse human B-cell populations, whether different stimulatory conditions induce lineage-distinct Breg subsets, or whether the directed methods used to characterize their phenotypes, that is, flow cytometry, have simply failed to identify an existing common pathway and underlying phenotype. In vitro stimulation can also alter cell surface phenotype and mask differences between expression profiles in Breg cells,^14^ limiting the ability of such approaches to accurately define Breg subsets following IL-10 induction.

Whole genome microarray analyses have attempted a more granular evaluation of human Breg phenotype^15,16^ (Figure 1C). Analysis of magnetic activated cell (MACS)–sorted IL-10^+^ and IL-10^−^ B cells following in vitro activation via Toll-like receptor (TLR) 9 ± B-cell receptor (BCR) stimulation, identified differentially expressed genes (DEGs) between these 2 subsets. Only one common DEG, encoding FCGR2B, a gene implicated in countering BCR-induced activation, was identified across the 2 studies, perhaps reflecting the different IL-10 induction protocols. Both studies identified that B cells at varying stages of differentiation, including naïve and memory B cells, could secrete IL-10 upon activation, with connectivity map analysis revealing that IL-10^+^ B cells induced by CpG oligodeoxynucleotide (CpG ODN) and immunoglobulin were differentiating towards a germinal center B-cell fate. These studies suggested that a distinct subset of transcription factors could be differentially expressed in IL-10^+^ human B cells, but that none of these were exclusively expressed in IL-10^+^ B cells.

Within such studies, phenotypic analysis is largely based on induced IL-10 expression rather than regulatory function, with the assumption that IL-10 is a specific functional marker for B cell–mediated immunoregulation.^9,10^ However, proinflammatory cytokines can be coexpressed with such stimulation, and the diverse spectrum of IL-10^+^ B cells identified may not be uniquely regulatory. Lighaam et al induced IL-10^+^ B cells in vitro using CpG ODN and anti-BCR stimulation and analyzed them by 12 predefined markers, using t-SNE to visualize high-dimensional flow cytometry data^9^ (Figure 1C). Glass et al utilized mass cytometry (CytTOF) to analyze expression of 24 surface and 14 intracellular proteins expressed by IL-10^+^ B cells following stimulation with CD40, TLR-ligand, and exogenous cytokine^10^ (Figure 1C). Neither approach identified phenotypes that uniquely defined Breg, but both studies reported a significant proportion of IL-10^+^ B cells, which coexpressed proinflammatory cytokines IL-6 and TNFα. IL-10 can support proinflammatory responses by preventing germinal center B-cell apoptosis and inducing plasma cell differentiation, antibody production, and IgG class-switching.^17-20^ Nevertheless, these observations may have clinical relevance despite the lack of a unique Breg signature. Cherukuri et al identified that the ratio of IL-10 and TNFα expressed by transitional B cells was a strong predictive biomarker of renal allograft outcomes including acute rejection and graft survival in human recipients.^5^ These findings underscore that it may be the balance of immunoregulatory versus proinflammatory B-cell parameters, which may be crucial to the overall clinical impact of Breg populations.

In summary, not all IL-10^+^ human B cells are regulatory. Different stimulation conditions can induce apparently phenotypically distinct B-cell subsets to express IL-10. By first phenotypically defining human Breg through IL-10-based stratification alone, or indeed other immunosuppressive mechanisms/markers in the case of IL-10-independent Breg such as Granzyme B and TGFβ,^21,22^ we are unlikely to reveal accurate and specific immunoregulatory signatures in human B cells.

### Alternative Approaches to Identify a Breg-specific Marker

Given the challenges outlined earlier, new strategies are needed to address Breg identity, as we move to translate the therapeutic potential of these populations to the clinic. Since we have no unique Breg identifiers, perhaps we should seek to define human Breg principally by their immunoregulatory function first, and then evaluate their markers second (Figure 1D).

Although humanized mouse models are available to interrogate function in a complex biological environment, these are not widely accessible. In contrast to murine Breg, the most common functional definitions of human Breg are established using a spectrum of ex vivo assays to evaluate the suppression of human immune responses.^1,13,14,23-26^ Commonly, such assays evaluate the impact of regulatory cell populations on proliferation and/or proinflammatory cytokine production by autologous T cells. These responses are usually driven by polyclonal stimulation with anti-CD3 and/or anti-CD28 beads, or by plate-bound anti-CD3 mAb, for 3 to 5 d.^1,13,14,23,24^ Crucially, such approaches have been validated in vivo, in humanized mouse models.^1^

Mechanistic context is important in the design of such experiments, however. Although CD40L-stimulated CD24^hi^CD38^hi^ Breg can suppress proinflammatory adaptive T-cell responses primarily via IL-10 secretion, they can also inhibit other cell types. The production of IFN-α by TLR9-stimulated plasmacytoid dendritic cells (pDCs), important drivers of innate and adaptive immune responses, can be suppressed by CD24^hi^CD38^hi^ Breg and thus used a measure of immunoregulation.^26^ Other groups have utilized TNFα expression by human monocytes to determine Breg function in the context of innate immunity.^14^ Apoptotic readouts within IL-2-stimulated T cells are required to assess the function of CD9^+^ Breg, which are known to drive T-cell cycle arrest and apoptosis in the context of severe asthma.^25^ The experimental question will dictate which suppression assay is most appropriate for functional interrogation of any particular Breg subset. Comprehensive phenotyping of functionally defined Breg can then be undertaken.

Bigot et al employed such a function-to-phenotype strategy by first isolating immunoregulatory CD19^+^CD24^hi^CD38^hi^, and nonregulatory CD19^+^CD24^int^CD38^int^ and CD19^+^CD24^hi^CD38^−^ B-cell subsets.^27^ These unstimulated subsets were analyzed by microarray analysis to reveal that CD19^+^CD24^hi^CD38^hi^ B cells displayed a distinct gene expression and cell surface marker profile when compared with their nonimmunoregulatory counterparts. Although these findings are promising, they are limited by the use of a microarray; this is a directed approach and novel transcripts cannot be detected because of the use of complementary probes.^28^ Dubois et al used RNA sequencing (RNAseq) to employ a more unbiased approach, analyzing immunoregulatory Granzyme B^hi^ B cells and nonregulatory Granzyme B^lo^ B cells^29^ to identify a molecular profile related to lymphocyte activation and regulation. However, this analysis was performed on bulk populations of purified B cells, which results in the averaging of distinct characteristics and can mask rare signals and variability.^28^

The emergence of single-cell RNA sequencing (scRNASeq) tools and complementary proteomic technologies such as CiteSeq, enables unbiased analysis of individual cells to decipher lineage hierarchies and cell states and provides an exciting strategy by which to characterize a human Breg-cell. Yang et al recently employed this technology to characterize mouse Breg by scRNASeq across multiple organs, delineating transcriptional profiles, and BCR clonotypes under physiological conditions.^30^ However, Breg were defined based on their expression profile of immunosuppressive and B10-related genes to define organ-specific, putative Breg subsets based on the score of B10 gene signatures. A strictly functional analysis by in vitro assays or in vivo models was not performed, and an assumption before analysis was made that all IL-10^+^ B cells were immunoregulatory.^9,10^ scRNASeq and CiteSeq have also been employed to define stimulated, expanded human Granzyme B^+^ Breg. DEG analysis identified 149 genes suggesting a promising transcriptional profile involved in proliferation, metabolism, decreased B-cell activation, and antigen presentation. However, the autologous Granzyme B^−^ B-cell population used for comparison was not stimulated, making it challenging to discern unique signatures of immunoregulation versus artifactual markers upregulated as a consequence of in vitro culture.^23^

These observations illustrate that key to such an analysis in human B cells will be the controls by which to compare functionally immunoregulatory B cells to nonregulatory counterparts. If the immunoregulatory B-cell population under analysis has been stimulated before single-cell analysis, then the comparative nonregulatory control populations should be similarly stimulated. A comprehensive and systematic analysis of known human B-cell populations enriched with functionally defined human Breg subsets, against similar yet nonimmunoregulatory B-cell populations, may provide a scheme by which to identify unique functional signatures of immunoregulation (Figure 1D).

## EXPANDING HUMAN BREG

**FIGURE 2.**
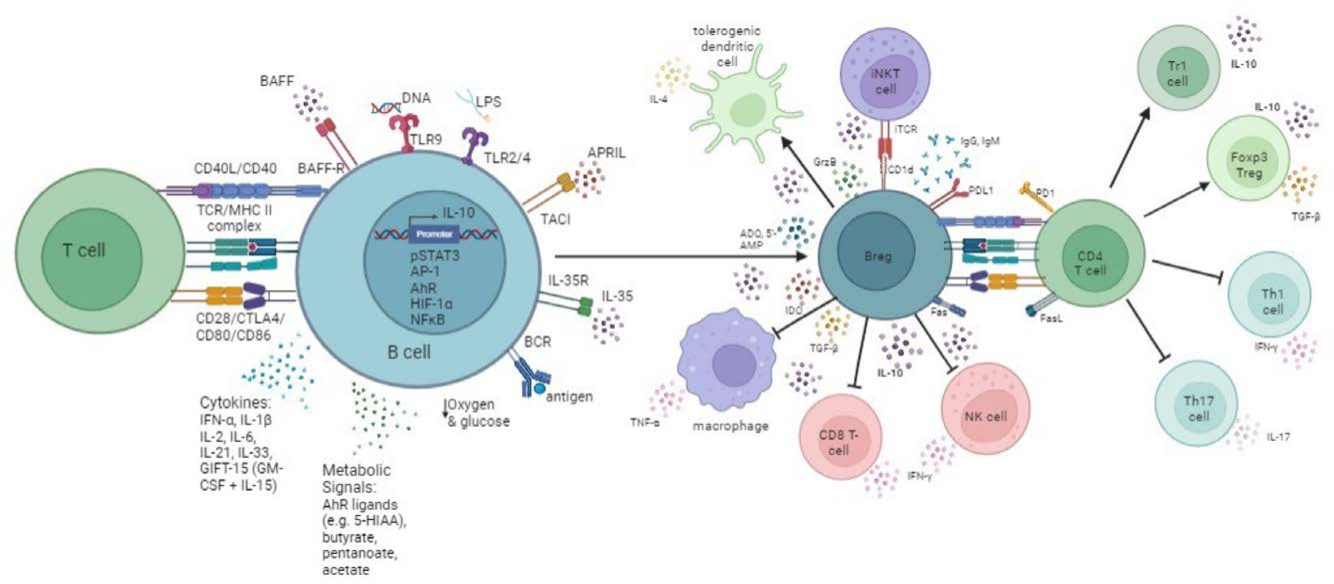
Breg can be induced through multiple mechanisms. To optimize development of human Breg-cell therapy, we need to determine the underlying principles and dynamics that drive human Breg induction, expansion, and functional stability. Understanding how different culture conditions and stimuli affects these parameters to generate diverse Breg subsets should provide a firm foundation for large scale applications. Multiple pathways may be harnessed to drive Breg expansion for cellular therapy. Figure generated in Biorender.

The use of human Breg as cellular therapy could be a potent option for transplantation and autoimmune disorders in a selected population of patients to minimize the use of nonspecific immunosuppression, reduce associated comorbidity, and improve long-term outcomes. To optimize development of human Breg-cell therapy, we need to determine the underlying principles and dynamics that drive human Breg induction, expansion, and functional stability (Figure 2). Understanding how different culture conditions and stimuli affects these parameters should provide a firm foundation for large-scale applications.

### Adaptive Signaling

#### The CD40^−^CD154 Interaction

Multiple studies have demonstrated the importance of CD40 ligation by CD154 (CD40 Ligand, CD40L) in the induction of most mouse and human Breg populations.^1,7,13,14,31-33^ The nature of engagement between CD40 and CD154 appears to be central in dictating human Breg differentiation.^34^ Relevant parameters include the form of CD40^−^targeting ligand, for example, soluble agonists such as monoclonal antibodies (mAbs),^32,35-37^ recombinant CD40L protein,^14,38^ or membrane-bound ligands such as those expressed by soluble membrane fragments^39^ and feeder cells^13,33,40,41^; ligand or agonist availability in terms of concentration or density^41,42^; ligand specificity for different CD40 epitopes.^43^ Different forms of CD40 stimulation in Breg induction protocols are summarized in Table 1. We have defined a Breg as a B cell, which demonstrates immunoregulatory function in an in vitro suppression assay and/or in an in vivo animal model.

**TABLE 1.** Ex vivo Breg induction ± expansion methods utilizing different forms of CD40 stimulation

CD40 stimulation	Species	B-cell source	Costimulus	Phenotype	Expansion factor	Disease process ± model tested	References
Anti-CD40 mAb	Mouse	Spleen	anti-IgM	CD23^hi^ CD21^hi^ CD24^hi^ CD93^+^ CD1d^hi^	NA	Lupus	^32^
Anti-CD40 mAb	Mouse	Spleen	BCR agonist	IL-10^+^	NA	EAE	^37^
Anti-CD40 mAb	Mouse	Spleen	LPS	CD5^+^ CD1d^hi^	NA	EAE	^44^
CD154^+^ NIH3T3 cell line	Mouse	Spleen	BLyS, IL-4, IL-21,	CD5^+^ CD1d^hi^	4x10^6^	EAE	^33^
Anti-CD40 mAb/Recombinant trimeric CD154	Human	Blood	CpG ODN	CD24^hi^CD27^+^	NA	RA In vitro assay	^14^
Recombinant trimeric CD154	Human	Blood	IL-21, IL-2, CPG ODN, anti-BCR mAb	GRZB^+^CD307b^hi^, GRZB^+^CD258^hi^CD72^hi^, GRZB^+^CD21^lo^PD-1^hi^	x30	Healthy donors In vitro assay	^23,24^
Recombinant trimeric CD154	Human	Blood	CpG ODN, IL-4	CD25^+^	NA	Healthy donors In vitro assay	^45^
Recombinant trimeric CD154	Human	Blood	CpG ODN	CD19^+^CD9^+^	NA	Asthma In vitro assay	^25^
Recombinant multimeric CD154	Human	Blood	IL-4	CD39^+^CD73^+^	NA	Healthy donors In vitro assay	^46^
CD154^+^ CHO cell line	Human	Blood	IL-2, IL-4, IL-10	CD73^−^ CD25^+^CD71^+^ TIM-1^+^CD154^+^	9x10^2^	Humanized mouse model of transplantation In vitro assay	^1^

Together with variable co-stimuli, such protocols induce diverse Breg subsets. Greater CD40 ligand (CD40L) availability encourages clustering of CD40 on the cell surface, leading to multimerization.

CHO, Chinese Hamster Ovary; EAE, experimental autoimmune encephalitis; RA, rheumatoid arthritis.

Extensive work by Neron’s group utilized the L4.5 cell line, a modified L929 cell line that expresses CD154 heterogeneously, to elegantly illustrate the importance of ligand availability in dictating downstream differentiation of human B cells. Stimulation by CD154^lo^ L4.5 cells maintained populations of CD19^+^CD27^+^ memory B cells and increased proportions of CD19^+^CD27^+^CD38^+^ B cells and CD19^+^CD138^+^CD38^++^ plasmablasts. In contrast, stimulation by CD154^hi^ L4.5 cells limited the CD19^+^CD27^+^ B-cell population, whereas promoting CD19^+^CD27^−^ naïve B-cell expansion.^41^ This differential effect has since been observed in the context of human Breg. Our group demonstrated 900-fold expansion of human CD19^+^IL-10^+^TIM-1^+^ Breg (expBreg), which exerted immunoregulatory function in vivo.^1^ expBreg prolonged human allograft survival in a humanized mouse model of skin transplantation and were able to induce human CD4^+^CD25^+^CD127^lo^ Treg.^1^ We identified that in varying the strength or availability of CD40^−^mediated stimulation by altering the concentration of CD154^+^ CHO cells, the rate of human IL-10^+^TIM-1^+^ Breg expansion and suppressive potency could be varied.^1^ The CD40^−^CD154 interaction acts as a rheostat, which can be used to direct B-cell differentiation. Increasing intensities of stimulation can fine-tune the differentiation of human B cells to result in different responses.^47,48^ These finding may help to explain the variety of human Breg subsets resulting from CD40^−^mediated stimulation (Table 1).^8^

Different anti-CD40 mAbs and CD40L display varying ability to induce proliferation and to activate different signaling pathways within B cells,^49^ likely due their differing epitopes and binding strengths.^43^ Iwata found that stimulation with soluble CD40L induced higher percentages of human B10 cells than CD40 mAb.^14^ Our group observed that CD40 mAb was unable to induce TIM-1^+^IL-10^+^ human Breg, even at high concentrations, unlike membrane-bound CD154.^1^ Quantifying CD40 signaling responses revealed a correlation between bound CD40L and degree of induced NFκB p65 phosphorylation, highlighting that CD40^−^signaling is a complex and highly regulated pathway.^50^ CD40^−^dependent rescue of human B cells from apoptosis requires minimal cross-linking and is essentially epitope independent. However, costimulus-independent CD40^−^mediated activation of resting B cells into the cell cycle related directly to the extent of CD40 receptor cross-linking.^51^ An alternative soluble agonist in the form of recombinant CD154, may provide a wider epitope selection as well a greater molecule density with the generation of trimeric^52,53^ and multimeric forms to generate human Breg in GMP protocols.^23,24,54^ A clear understanding of this differential signaling will help to develop feeder-cell-free coculture methods to ex vivo expand human Breg for cellular therapy in use in the clinic (Table 1).

#### BCR Signaling

Experimental studies have demonstrated that BCR signaling is important in both generation and function of Breg. BCR engagement is crucial for the development of murine B10 cells, with a 90% reduction observed in mice expressing an irrelevant antigen marker.^55,56^ Adoptive transfer of TIM-1^+^ splenic B cells from BALB/c tolerant mice prolonged BALB/c pancreatic islet graft survival in WT and B cell–deficient mice (µMT^−/−^), but not graft survival of third party C3H grafts.^57,58^ Mechanistic studies examining stromal interaction molecule (STIM) proteins and their control of intracellular calcium demonstrated that BCR signaling pathways can drive IL-10 expression and suppressive function, suggesting a potential strategy to selectively expand antigen-specific Breg.^59^ Breg therapy expansion protocols must therefore account for BCR stimulation or antigen recognition in its design and development.

### Innate Signaling

Although adaptive signals are important for Breg development and induction, innate signals also induce and expand Breg populations.^60^

#### TIM-1

The T-cell Ig and mucin domain 1 (TIM-1) receptor, a phosphatidylserine receptor, was first identified to be expressed on the majority of IL-10^+^ B cells, by Ding and colleagues.^61^ In this landmark study, the authors demonstrated that TIM-1 is expressed by the majority of IL-10^+^ B cells in all major subpopulations, including transitional, marginal zone, and follicular B cells. The binding of TIM-1 with a low-affinity anti-TIM-1 mAb can induce TIM-1^+^ Breg. Adoptive transfer of TIM-1^+^ Breg conferred allograft tolerance in mouse models of islet transplantation.^61^ Subsequent studies demonstrated that B cells, which express a mutant form of TIM-1 (TIM-1(Δmucin)) lacking the mucin domain, exhibited decreased phosphatidylserine binding, and were unable to produce IL-10 in response to apoptotic cells or by specific ligation with anti-TIM-1 mAb. TIM-1(Δmucin) mice demonstrated accelerated allograft rejection, which appeared to be due in part to deficiencies in both baseline and induced IL-10^+^ Breg.^62^

Our group demonstrated that the importance of TIM-1 expression in human Breg. TIM-1 knockout experiments showed that expBreg were dependent on TIM-1 to modulate STAT3 phosphorylation and control suppressive potency.^1^ When phosphorylated upon activation by a variety of stimuli including IL-10, IL-21, and CD40L, pSTAT3 translocates from the cytoplasm to nucleus, where it binds to the IFN-γ–activated sequence in target promoters and activates transcription, including that of IL-10.^63^ The importance of this pathway in Breg function is illustrated in autoimmune disease. CD19^+^CD24^hi^CD38^hi^ B cells from patients with systemic lupus erythematosus demonstrated reduced STAT3 phosphorylation in response to CD40 engagement together with reduced IL-10 expression and in vitro suppressive function, relative to healthy individuals.^13^ The inclusion of TIM-1-specific ligands within Breg expansion cultures may further enhance suppressive potency and proliferation of a future cell therapy product.

#### TLR Signaling

Multiple studies have reported Breg induction via TLR binding in humans and mice, including CpG ODN,^64^ bacteria,^65^ and parasites.^66^ The impact of TLR-mediated signaling in Breg populations is demonstrated in a microbiota-mediated immune tolerance model; germ-free mice colonized with wild-type microbiota generated CD5^+^CD1d^hi^CD21^hi^CD23^−^IL-10^+^ intestinal B cells, which shared phenotypic features with splenic marginal zone B cells and B10 cells.^67,68^ TLR ligands and enteric bacterial lysates preferentially induced IL-10 production and regulatory function of intestinal B cells, which ameliorated chronic T cell–mediated colitis via TLR2, MyD88, and PI3K-dependent pathways.^68^ MyD88 is an adaptor protein common to all TLRs and may be a therapeutic focal point for TLR-induced Breg subsets.^69^ MyD88-deficient mice demonstrate reduced frequency of splenic B10-like IL-10^+^ Breg compared with wild-type mice following lipopolysaccharide (LPS) stimulation.^55^ IL-10 production by TLR-stimulated B cells increased upon CD40 engagement, reinforcing the observations that regulatory function in phenotypically similar B cells can be induced utilizing multiple signaling pathways to augment suppressive potency.^70,71^

Other innate stimuli may induce similar Breg responses; ex vivo stimulation of mouse splenic mononuclear cells with LPS and Astilibin induced both CD19^+^CD1d^hi^ and CD19^+^TIM-1^+^ Breg in STAT3-dependent mechanisms.^72^ Notably, Breg induction was weakened in CD40^−/−^ B cells with a decrease in STAT3 activation, whereas induction was abrogated in TLR4^−/−^ B cells with no STAT3 activation. Conversely, induction and proliferation of phenotypically different Breg populations can be controlled by stimuli acting through common intracellular pathways. TGF-β-dependent and TIM-1-enriched splenic Breg could be induced by a combination of CpG ODN and LPS, PMA and Ionomycin to prevent allograft rejection by an IL-10-independent mechanism.^73^

Certain stimuli may promote particular Breg subsets, whereas inhibiting others. Curcumin can inhibit the TLR/MyD88 pathway to treat ulcerative colitis in an experimental murine model. Curcumin upregulated CD19^+^CD1d^+^, CD19^+^CD25^+^, and CD19^+^Foxp3^+^ Breg, whereas downregulating CD19^+^PD-L1^+^, CD19^+^TIM-1^+^, and CD19^+^CD27^+^ Breg.^74^ Careful consideration will be required when selecting adjuncts for ex vivo Breg expansion, to ensure selection of the target Breg population for clinical expansion. Identification of robust human Breg phenotypes as outlined in the first section is required for the latter, whereas systematic preclinical optimization and process development, discussed in the following section, will be needed to achieve the former.

## METABOLIC PATHWAYS

Control of immune cell metabolism is critical in regulating fundamental immunological processes. There is growing evidence to suggest that metabolic factors are a major contributor to Breg induction.

### Aryl-hydrocarbon Receptor

Rosser et al identified that reduced levels of butyrate in the gut were associated with reduced frequency of CD19^+^CD24^hi^CD38^hi^IL-10^+^ Breg in peripheral blood of rheumatoid arthritis patients. Butyrate supplementation ameliorated disease in wild type but not B cell–deficient mice with arthritis and improved suppressive potency of IL-10^+^CD19^+^CD21^hi^CD24^hi^ Breg.^75^ Butyrate supplementation increased the level of the serotonin-derived metabolite 5-hydroxyindole-3-acetic acid (5-HIAA), which activated the aryl-hydrocarbon Receptor (AhR), which then localized to a transcription start site upstream of the IL-10 locus to promote IL-10 expression.^75,76^ Transcriptome analyses demonstrated that loss of AhR in B cells reduced IL-10 expression by skewing the differentiation of CD19^+^CD21^hi^CD24^hi^B cells into a proinflammatory program, under Breg-inducing conditions.^76^

### Cholesterol Metabolism

Synthesis of the metabolic intermediate, geranylgeranyl pyrophosphate (GGPP), is required to drive IL-10 production in Th1 cells and to attenuate Th1 responses.^77^ Bibby et al demonstrated a critical role for cholesterol metabolism via the geranylgeranyltransferase (GGTase)-GGP axis, in supporting human Breg function through the control of IL-10 expression in human CD24^hi^CD27^+^ and CD24^hi^CD38^hi^ B cells following TLR9 stimulation.

The clinical impact of such signal dysregulation can be appreciated in disorders, which involve mutations in this metabolic pathway: patients with mevalonate kinase deficiency develop severe and recurring autoinflammatory fevers, associated with dysregulated B-cell responses including elevated serum immunoglobulin levels.^78^ Patients with mevalonate kinase deficiency demonstrated reduced frequency of CD24^hi^CD38^hi^ B cells and poor IL-10 responses across all B-cell subsets when compared with healthy individuals. Addition of GGPP could reverse defects in IL-10 production.^79^ Isoprenyl modifications may constitute a metabolic preprogramming event, requiring the generation of GGPP before cellular stimulation to fine-tune signaling cascades. The state of signaling intermediates may be predetermined by the state of cholesterol metabolism of the quiescent cell. Controlling for such parameters may be crucial in ensuring optimal precursor selection and/or priming of human B-cell subsets before ex vivo expansion for cell therapy.

### Hypoxia-inducible Factor-1α

The transcription factor hypoxia-inducible factor-1α (HIF-1α), a major regulator of oxygen homeostasis within cells, can regulate IL-10 transcription and promote expansion of murine CD1d^hi^CD5^+^ B10 cells through precise control of the glycolytic pathway. Mice with HIF-1α-deficient B cells demonstrated reduced frequency of B10 cells and developed exacerbated disease in experimental autoimmune encephalitis (EAE) and collagen-induced arthritis (CIA) models. Notably, this effect could be reversed in mice transfected to overexpress IL-10. HIF-1α coprecipitated with STAT3 under hypoxic conditions and associated with the hypoxia-responsive element I and hypoxia-responsive element II regions of the IL-10 promoter, providing a mechanism by which low oxygen levels and/or glucose metabolism within an inflammatory microenvironment could influence Breg differentiation and expansion.^80^ Although parallel mechanisms are yet to be characterized in human Breg, the recent advent of HIF-1α modulators pose tantalizing therapeutic possibilities to manipulate Breg function.

Thus, similar to TLR and CD40^−^signaling, metabolic pathways may be able to act in synergy or specifically in response to unique stimuli and underlying metabolic status, to promote Breg induction and function. Such insights may be utilized when optimizing protocols for clinical therapeutic use.

## CYTOKINE SIGNALING

Cytokines are involved in both adaptive and innate immune pathways and have proven critical to the induction and expansion of mouse and human Breg subsets.^1,24^ Combinations of such adjuncts may improve potency and stability of an ex vivo expanded Breg-cell therapy.

### IL-1β, IL-6, IFN-α

Rosser et al described Breg induction by proinflammatory cytokines IL-1β and IL-6, as a method of autoregulation in CIA.^81^ Antibiotic treatment limited CIA: the loss of commensal bacteria altered stimulation of macrophages and dendritic cells within gut-associated lymphoid tissue, resulting in reduced IL-6 and IL-1β expression. These observations were associated with decreased frequency in splenic IL-10^+^ Breg, an effect replicated in IL-6R^−^ and IL-1β^−^ mice. Stimulation of naïve splenic B cells with IL-6, IL-1β, and anti-CD40 mAb-induced expansion of all splenic Breg subsets including IL-10^+^ transitional 2-marginal zone precursors (T2 MZP), IL-10^+^CD5^+^ cells, IL-10^+^TIM-1^+^ cells, and IL-10^+^ B10 cells. Moreover, this combined stimulation resulted in greater levels of phosphorylation in NF-κB and STAT3 compared with either cytokines or CD40 mAb alone.

Similarly, Menon et al demonstrated a regulatory feedback loop between plasmacytoid dendritic cells (pDCs) and Breg.^26^ In healthy human subjects, pDCs were able to drive the differentiation of IL-10^+^CD24^hi^CD38^hi^ Breg and plasmablasts via the release of proinflammatory cytokine, IFN-α, and the engagement of CD40, in vitro. CD24^+^CD38^hi^ Breg cells subsequently restrained IFN-α production by pDCs via IL-10, providing a feedback mechanism of regulation. This interaction was compromised in the context of systemic lupus erythematosus, where pDCs promoted plasmablast differentiation but failed to induce Breg. Defective pDC-mediated expansion of Breg was associated with altered STAT1 and STAT3 activation. In support of these autoregulatory cycles of Breg induction, Breg frequency increases during the inflammatory phase of several autoimmune disorders.^14,37,82,83^

### IL-35

IL-35 is an immunosuppressive member of the IL-12 cytokine family, and can suppresses CD4^+^ T-cell proliferation and promotes regulatory T-cell (Treg) expansion. IL-35^+^ B cells were able to profoundly suppress inflammation in mouse models of experimental autoimmune uveitis and EAE, and in some instances release IL-35-containing exosomes.^84-86^ CD5 and TIM-1 expression was upregulated in IL-35^+^ Breg and recombinant IL-35 could induce IL-35^+^ Breg mediated through the STAT1/STAT3 pathway.^84^ Crucially, IL-35 induced the conversion of human B cells into Breg cells, suggesting that IL-35 may be used to induce autologous Breg.^85^ Reduced IL-35 expression also correlated with greater disease activity in ankylosing spondylitis patients. Stimulation of PBMCs from these patients with recombinant IL-35, resulted in the induction of IL-10^+^ CD19^+^CD24^hi^CD38^hi^ Breg,^87^ demonstrating that rIL-35 may be a useful stimulant within ex vivo expansion protocols to generate a spectrum of Breg populations.

### IL-21

First described as a potent inducer of the murine B10 subset, IL-21 could increase both Breg frequency and IL-10 expression in ex vivo cultures, whereas being essential for both B10 expansion and suppressive function in an EAE mouse model.^33^ Our group has demonstrated a similar phenomenon in ex vivo expanded human IL-10^+^TIM-1^+^ Breg. IL-21 stimulation increased human Breg suppressive potency and STAT3 phosphorylation.^1^ Studies in cancer have demonstrated that Granzyme B^+^ Breg can be induced in vitro by IL-21 and BCR stimulation of human CD19^+^ blood cells, express key regulatory markers including IL-10, CD25, and indoleamine-2,3-dioxygenase and suppressed T-cell proliferation by granzyme-B-dependent degradation of the T-cell receptor ζ-chain.^88^ Recently, Chesneau et al was also able to in vitro expand granzyme B^+^ Breg using an expansion cocktail including IL-21, anti-BCR, CpG ODN, CD40L, and IL-2.^24^ Similar to IL-35, IL-21 is likely to be an important component of ex vivo expansion protocols to generate both human IL-10-dependent and IL-10-independent Breg.

### BAFF, TACI, and APRIL

The TNF family ligands, A proliferation-inducing ligand (APRIL) and B cell activating factor (BAFF), are known to support B-cell survival and to drive Breg expansion.^33,89-91^ These factors interact with 3 TNFR family members, Transmembrane activator and CAML interactor (TACI), BAFF-R, and B-cell maturation antigen (BCMA) with differing affinities. BAFF-R and TACI demonstrate the highest affinity for BAFF and APRIL, respectively.^92^ Differential receptor binding may in part account for the seemingly diverse Breg populations generated by these 2 ligands.

Stimulation of human CD19^+^ B cells with APRIL, IL-21, and CD40 preferentially generated a novel IL-10^+^IgA^+^ Breg subset which exhibited upregulated expression of FasL and PD-L1^89^. Stimulation of human PBMC with CpG ODN and soluble APRIL, rather than BAFF, expanded human B10 cells in an APRIL-dependent manner. TACI expression was greater in B10 cells than IL-10^-^ B cells, whereas BAFF-R expression was lower.^89^ Notably, B10 cells from patients with rheumatoid arthritis were responsive to APRIL, suggesting a possible therapeutic application of APRIL to expand human B10 cells. BAFF can induce IL-10^+^ Breg in mice and support human Breg survival.^90,93^ Human PD-L1^hi^ Breg have been shown to preferentially sequester BAFF via BAFF-R, eliciting a potential survival advantage.^90^ However, loss of immunoregulatory capacity of human precursor marginal zone Breg appears to be directly related to an excess of BAFF in the context of HIV-1 infection.^94^ Careful evaluation of these B-cell survival factors in ex vivo protocols may improve the expansion and viability of a human Breg-cell therapy product.

## SCALING BREG-CELL CULTURE FOR CELL THERAPY

The role of Breg in transplantation and autoimmunity has led to a multitude of Breg culture protocols as outlined above. However, Breg expansion has been so far limited to a laboratory setting. Within a clinical context, additional variables must be considered for prospective therapies: depending on the size of the patient, the cell dose may vary; media formulations and stimulants are tailored to cell type^95^; environmental factors must be maintained and account for duration of culture and culture vessel used.^96^ It will be necessary to have efficient manufacturing technologies and standardized GMP protocols in place.

## CULTURE SYSTEM

A primary concern when scaling cell therapies is choosing an appropriate growth vessel. Culture systems are selected based on several factors including required yield, manufacturing costs, and possibility for full or semiautomation.

### Small Scale and Static Culture

Treg cell therapies, applied in similar clinical contexts as Breg, are currently limited to small-scale production methods, which can be costly relative to output.^97,98^ Chimeric antigen receptor (CAR) T cells in contrast make use of a combination of static culture vessels for small-scale expansion and dynamic bioreactors,^99^ usually incorporating a perfusion regime to replenish media and stimulants.^100^ The Gas-Permeable Rapid Expansion (G-Rex) system^101^ consists of a cell culture vessel with a gas-permeable membrane at the bottom for ease of gas exchange. These flasks can be incubated in laboratory cell culture incubators. It is considered a practical and cost-effective way to scale up CAR T-cell cultures, as it does not require additional specialized equipment or sensors. However, the lack of automation and small-scale nature of static culture systems leave manufacture vulnerable to inefficiencies and contamination.

### Larger-scale Bioreactors

Rocking motion bioreactors, such as the GE WAVE bioreactor system, enable larger-scale cultures in a semiautomated process.^101^ These systems employ rocking platforms, which maintain inflation of culture bags containing the cells, whereas providing sufficient mixing for improved gas exchange and homogeneity of nutrients required for cell expansion. In contrast to small-scale and static culture conditions, these systems can incorporate fluid filtration systems to retain cells, whereas replacing stimuli and media, thus limiting the need for additional preclinical purification.^102^ Drawbacks include the possibility of mechanical failures and contamination because of the semiautomated technology.

### Closed Systems

Cell therapies should ideally aim for closed system expansion protocols without the need to replace components. Efficient, highly controlled, and automated closed-system technologies can reduce labor-intensive procedures, eliminate the possibility of contamination and improves GMP compliance.^96,101,103^ The CliniMACS Prodigy (Miltenyi Biotec) and the Cocoon (Lonza), both fully automated expansion technologies, have been used to manufacture CAR T cells; they involve one-system, multistep technologies that integrate and automate the process to achieve high yields and high reproducibility through a standardized workflow.^104,105^

**FIGURE 3.**
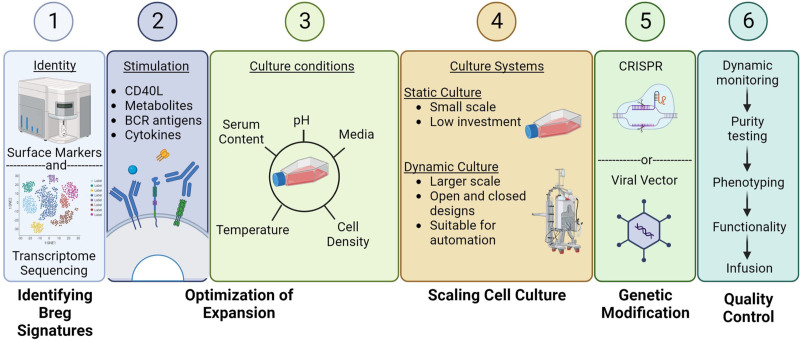
Outline of process development to translate Breg from the bench to the bedside. The definition of unique Breg identity may provide insights in optimal Breg precursor selection for expansion, mechanistic pathways, which can boost suppressive function and surrogate markers by which to analyze the quality of a Breg-cell therapy product. Systematic evaluation of potential stimulatory adjuncts including cytokines and feeder cells, together with the culture conditions required for optimal expansion will inform which culture system is most appropriate. Ancillary modifications including genetic editing may enhance Breg potency and stability. Robust quality control procedures will be required to ensure safety and efficacy before administration to a transplant recipient. Figure generated in Biorender.

Breg expansion protocols have specific requirements that may dictate which large-scale cell culture systems can be used. The required strength of CD154 stimulation is most reliably induced via cell-cell interactions. However, this may be limiting and labor intensive, as it introduces a feeder cell that may require repeated replenishment and subsequent exclusion before use in the clinic. An alternative could make use of microcarriers in suspension, an approach already applied for vaccine production. This method makes use of small particles or beads coated in a protein of choice to provide stimulation.^106^ Similarly, the use of multimeric CD154 Abs or ligands may provide sufficient stimulation to induce and expand human Breg ex vivo.^107^ Recently, Goodall et al generated a virus-like particle (VLP) with tunable expression of CD154.^108^ Quantification of multivalent display of CD154 by the VLP demonstrated that higher saturations of bacteriophage corresponded with greater cellular signaling, resulting in a VLP with modifiable expression of CD154. Such a tool could be repurposed to precisely control human B cell stimulation and Breg expansion in this context.^109^ These modifications may enable the utilization of fully automated, closed systems and should be explored as part of process development (Figure 3).

## CULTURE COMPONENTS

The components required for successful Breg culture also require careful thought when designing a system for clinical application (Figure 3).

### Media

Most laboratory-based Breg expansion protocols rely on commercially available media formulations, which are typically supplemented with calf serum. Work with T-cell therapies has demonstrated the considerable effects that different cell culture media can have on expansion and cell differentiation.^110^ Sato et al found that the use of OpTmizer media resulted an improved T-cell expansion rate and effector functions compared with RPMI and AIM-V.^111^ Gene expression analysis of effector molecules and T-cell cytotoxicity function were higher when cultured in OpTmizer than in AIM-V media, with the latter showing lower expression levels compared even with control PBMC. When comparing AIM-V and TexMACS media, it was observed that TexMACS promoted the expansion of T effector cells, whereas AIM-V promoted the expansion of T central memory cells. Such progress in other immunotherapy domains illustrates the need for meticulous assessment of the most elementary of cell culture components when optimized human Breg expansion protocols.

### Serum

Pooled human serum (HS) from blood group AB donors is generally considered a preferred alternative to xenogenic, fetal bovine serum for therapeutic purposes.^100^ However, as with fetal bovine serum, HS lacks consistency because of lot variability. Serum of either source can also be a rate-limiting factors in CAR T-cell expansion during upscaling.^112^

Exploration of different kinds of serum have demonstrated variability in transduction efficiency of the CAR gene for CAR T-cell therapy. The development of advanced serum-free medium formulations for expansion of cell therapy have had varying degrees of success in growth rate.^112,113^ Recent developments of serum alternatives can improve the growth kinetics and efficiency of expanded cells, whereas reducing batch variability. Human platelet lysate (hPL), produced from transfusable human platelets, and PhysiologixTM xeno-free hGFC (Phx) are known HS alternates.^114-116^ The addition of hPL has exhibited comparable expansion rates to serum-supplemented cultures to generate T effector cell immunotherapies in the context of cancer but also maintains comparably higher percentages of T central memory cells. The potential effects of serum and available substitutes are yet to be rigorously explored in the context of human Breg expansion and will form an important aspect of process development.

### Cytokines and Pharmaceutical Adjuncts

Most Breg induction/expansion methods require soluble signals to induce expansion, many engaging common transcription pathways. These stimuli may skew human Breg expansion to different phenotypes.^33,81,87,89^ Large-scale cell culture will benefit from detailed characterization of which combinations of stimuli will generate the desired human Breg phenotype and from which optimal human B-cell subset precursors, alongside duration of culture.^117^ Elucidation of such approaches and utilization of pharmaceutical compositions including those to modulate HIF-1α, STAT3 and TLR/MyD88 pathways, Breg metabolism, and IL-10 expression may provide a basis for optimal ex vivo expansion of autologous human Breg-cell therapy.

## GENETIC MODIFICATIONS

Preclinical work has demonstrated the improved efficiency of alloantigen-specific Treg when compared with polyclonal Treg.^118,119^ Phase 1 clinical trials of CAR-Treg therapy in renal transplantation are now underway.^120^ Antigen-specific B cells have a low precursor frequency and are difficult to isolate and expand in high yields.^121^ The advent of CARs is revolutionizing T-cell therapy and could facilitate development of an antigen-specific IL-10^+^ Breg therapy.^122^ CAR structures to support human Breg therapy could focus on signaling pathways strongly associated with suppressive function and functional stability, such as TIM-1, STAT3, IL-10, and TLR/MyD88.

Genetic editing of primary human B cells is challenging, but several different technologies are now emerging with promising effects in this lymphocyte population. Lessons can also be learned from murine B cell-based strategies. Early attempts used a retroviral vector to encode B cells with an antigen fused to an immunoglobulin heavy chain, which successfully inhibited autoimmunity in mice following adoptive transfer.^123^ However, as activated B cells may risk inflammation, quiescent or resting B cells have also been targeted, with Calderón-Gómez et al transducing cells to express either IL-10 or a disease relevant autoantigen.^124^ Moffet et al incorporated both an AAV-transfection method together with CRISPR-based genetic editing to replace the variable regions of B-cell immunoglobulin with a target of choice, effectively altering the antigen specificity of a human B cell.^125^ Lentiviral approaches have also been reported in mouse and human B cells,^126^ offering a multitude of different gene editing approaches by which to modify human Breg and to incorporate such strategies into scalable culture systems.

## CONCLUSION

Rigorous evaluation of Breg over the last decade in mouse and human models, together with advances in single-cell technologies and closed culture systems, have set the stage for the optimization of Breg cellular therapy, clarification of Breg identity, and the development of dependable biomarkers. Although we are inching closer to clinical translation, a robust framework will be needed by which to benchmark Breg for administration in a transplant recipient. Such a system will require specific Breg markers which are indicative of function to facilitate the assessment of quality, yield, and stability of any human Breg product before clinical use. Systematic evaluation of Breg precursor populations, expansion stimuli and optimal culture systems for large-scale application, will provide the bridge to translation. Whether applied as an adjunct to minimized immunosuppression regimens or as part of a multi-regulatory cell therapy cocktail, we are close to realizing the potential of this powerful arm of the immunoregulatory system to improve clinical outcomes for our transplant recipients.
